# Two-tier charging in Maputo Central Hospital: Costs, revenues and effects on equity of access to hospital services

**DOI:** 10.1186/1472-6963-11-143

**Published:** 2011-06-02

**Authors:** Barbara McPake, Charles Hongoro, Giuliano Russo

**Affiliations:** 1 Institute for International Health and Development, Queen Margaret University, Queen Margaret University Drive, Musselburgh, EH21 6UU, UK; 2 Health Systems Research Unit, South African Medical Research Council, 1 Soutpansberg Road, Pretoria Private Bag ×385, 0001 Pretoria, South Africa; 3 International Public Health and Biostatistics Unit, and Centro de Malária e outras Doenças Tropicais, Instituto de Higiene e Medicina Tropical, Universidade Nova of Lisbon. Rua da Junqueira 100, 1349-008 Lisbon, Portugal

## Abstract

**Background:**

Special services within public hospitals are becoming increasingly common in low and middle income countries with the stated objective of providing higher comfort services to affluent customers and generating resources for under funded hospitals. In the present study expenditures, outputs and costs are analysed for the Maputo Central Hospital and its Special Clinic with the objective of identifying net resource flows between a system operating two-tier charging, and, ultimately, understanding whether public hospitals can somehow benefit from running Special Clinic operations.

**Methods:**

A combination of step-down and bottom-up costing strategies were used to calculate recurrent as well as capital expenses, apportion them to identified cost centres and link costs to selected output measures.

**Results:**

The results show that cost differences between main hospital and clinic are marked and significant, with the Special Clinic's cost per patient and cost per outpatient visit respectively over four times and over thirteen times their equivalent in the main hospital.

**Discussion:**

While the main hospital cost structure appeared in line with those from similar studies, salary expenditures were found to drive costs in the Special Clinic (73% of total), where capital and drug costs were surprisingly low (2 and 4% respectively). We attributed low capital and drug costs to underestimation by our study owing to difficulties in attributing the use of shared resources and to the Special Clinic's outsourcing policy. The large staff expenditure would be explained by higher physician time commitment, economic rents and subsidies to hospital staff. On the whole it was observed that: (a) the flow of capital and human resources was not fully captured by the financial systems in place and stayed largely unaccounted for; (b) because of the little consideration given to capital costs, the main hospital is more likely to be subsidising its Special Clinic operations, rather than the other way around.

**Conclusion:**

We conclude that the observed lack of transparency may create scope for an inequitable cross subsidy of private customers by public resources.

## Background

'Private', 'high-cost' and 'special' clinics and wards are increasingly common features of public hospitals in Africa and other parts of the world [1,2]. These services are targeted at middle and upper class groups who are prepared to pay higher charges for services with higher levels of amenity (such as hotel services which are not considered clinically relevant), or in some cases for choice of doctor [1]. A common rationale for the development of such services is that they will generate resources for under-funded parts of the health system. For example in some Provinces of South Africa, the policy is being developed with the objective of generating resources largely for the primary care system [3]. In other settings, for example Zambia, resources are retained at hospital level with the objective of generating resources for services targeted at the majority of the population [4].

However, the policy is not universally endorsed. It inevitably creates inequity by creating two-tier service provision. It may be difficult to maintain a quality separation between the two services based only on amenity levels, and to ensure comparable clinical quality of care [1,2]. Equally difficult to establish is the balance of net resource flows between services. Accounting systems are seldom set up to enable this. Private clinic and ward users access services from all over the hospital where there is no dedicated service. Resources generated through private services are seldom lodged in a single profit and loss account and then distributed. Financial transactions are complex. No study that we know of has traced resource flows through the full breadth of a hospital operating a two-tier charging strategy with the objective of identifying net resource flows between the two services. This was the objective of the study reported in this paper.

In Mozambique, the existence of dual practice is recognised with both public sector physicians owning private clinics and others providing private services within central, provincial, general and, to some extent rural hospitals - the so-called "special services" and "special clinics" [5]. At the time of the study, Maputo Central Hospital (MCH) was the largest hospital in Mozambique with 1,518 beds and about 2,000 employees. The creation of the MCH Special Clinic was authorised in 1977 after independence by the FRELIMO ruling party and then regulated by the Government through successive legislation, with the objective of providing adequate care for party members and international diplomatic officials in Maputo [6,7]. According to the original regulation, selected venues of the MCH were to be rehabilitated for the purpose, and MCH staff was to serve in the special clinic outside their regular shift for extra compensation. Since 1977 the special clinic has grown to include 4 departments, 36 beds and employs 71 full time staff, for an annual turnover of about 1 million USD. The special clinic offers all the services available in the main hospital, offering patients the advantages of choice of physicians and time of visit, booking service, and attendance in separate facilities. The majority of special clinic activities take place in the restricted premises assigned to the clinic, with the exception of surgical operations and laboratory analysis, for which the special clinic makes use of the main hospital facilities in exchange for direct payments to personnel and departments involved. Heads of department and selected medical staff from the main hospital serve in the special clinic. Nurses and non-medical staff from the main hospital rotate annually to work a regular shift in the special clinic. The special clinic pays a monthly sum of 200 million MZM (about 10,000USD at the time of the study) to the main hospital as a contribution to the MCH general expenses.

In MCH, the special clinic sits uncomfortably adjacent to the accident and emergency department. This means that critically ill patients waiting long periods for services in hot and dusty conditions can view richer hospital users with more minor conditions securing fast access to service in comfortable conditions. This makes the two-tier service unpopular and politically conspicuous. Since the 2005 election the new FRELIMO government signalled its intention to abolish the institution of Special Clinics across the country, and a new minister from the MCH was put in charge to reform the system under a highly publicised anticorruption agenda [8]. In 2007, a ban on private practice within hospital facilities was introduced, to eliminate special services and special clinics within the country's major hospitals, although still allowing publicly employed physicians to practice in private clinics outside their public hospital hours [9]. With a sudden change of leadership in 2011, the debate around the MCH Special Clinic appears to have died out.

To date, a few costing studies [10,11] have been carried out focusing on specific parts of the hospital but this was the first to cover MCH as a whole and to include costing of the Special Clinic.

## Methods

Data were collected and analysed during 2001, pertaining to the financial year 2000-2001.

### Step-down costing

Most cost items were estimated using a step-down costing method [12]. This method uses data available at hospital level and disaggregates by direct cost centre and indirect cost centre. A direct cost centre is a unit within the hospital that directly produces the output of interest, for example inpatient or outpatient stays and visits. An indirect cost centre is a unit, which does so indirectly, for example by producing diagnostic test results, which are the constituent components of an outpatient, stay or visit.

Costs were apportioned using the apportionment criteria described in Table 1, in some cases via an intermediate cost centre. Intermediate cost centres that were themselves direct consumers of resources were apportioned to final cost centres using the criteria described in Table 2.

**Table 1:** Cost categories and apportionment methods

Cost category	Indirect cost centre (where applicable)	Final apportionment
*Non-salary personnel expenses*		
Per diems	Administration	Inpatient equivalents
Funeral subsidy		Total salary cost

*Goods*		
Fuel and lubricants	Transport and logistics	Number of admissions
Infrastructure maintenance		Floor space
Equipment maintenance		Equipment value
Perishable stationery	Administration	Inpatient equivalents
Uniform and shoes		Total staff numbers
Other perishable goods	Administration	Inpatient equivalents

*Services*		
Communications	Administration	Inpatient equivalents
Flight tickets	Administration	Inpatient equivalents
Facility rent	Administration	Inpatient equivalents
Infrastructure maintenance		Space
Equipment maintenance		Equipment value
Transport and cargo	Transport/logistics	Admissions
Insurance	Administration	Inpatient equivalents
Legal representation	Administration	Inpatient equivalents
Consultancy and technical assistance	Administration	Inpatient equivalents
Water and electricity		Floor space
Health information	Administration	Inpatient equivalents
Other services		Floor space

Current transfers		
Customs tax	Administration	Inpatient equivalents

**Table 2:** Apportionment method used for intermediate cost centres

Intermediate cost centre	Apportionment
Central operating theatre	Number of operations

Blood	Consumption of blood units

Pathology	Pathology investigations ordered

Clinical analysis	Laboratory tests ordered

Imaging	Images requested

Pharmacy	Drug consumption

Food provision	Patient and staff days

Laundry	Patient days

Hygiene and cleaning	Floor space

Mortuary	Admissions

Transport and logistics	Admissions

Administration	Inpatient equivalents

### Bottom-up costing

Step-down costing was complemented by selective use of bottom-up costing for the specific major inputs, staff, drugs and medical supplies, and capital items [13]. The choice and combination of methods depended on the nature and estimated reliability of data at different points in the hospital and are described for each of the main cost categories.

#### Staff costs

Number of staff by grade, department and/or service unit, permanent or contract was established by visiting all department heads. Salary mid-points and average or standard allowance packages were calculated for each staff grade. These combined to produce estimates of annual total staff costs for each service unit or department as appropriate. At the special clinic, aggregate annual personnel costs for both permanent and contract workers were obtained from the financial statement and from service specific cost records. Discrepancies were found between the two sources and the aggregate financial statement was apportioned according to the shares of staff costs in the service specific cost records.

#### Drug and medical supplies costs

A centralised and computerised system of drugs distribution and consumption by service units was used at MCH. Price data were obtained from government tender documents used in drug and medical supply procurement, and from private pharmacies where those were not available. The hospital pharmacy did collate patient origin - inpatient or outpatient - data. A review of dispensing records for the previous 3 weeks was carried out to estimate apportionment factors. Like drugs, medical supplies are sourced from Central Medical Stores but there is a specific warehouse that stores and distributes all types of medical supply (but not surgical supplies) once received by the hospital. The central operating theatre stores and distributes surgical supply. The medical supplies warehouse keeps stock records. A randomly selected 3-month sample of these was reviewed in order to apportion medical supplies costs. For surgical supplies, the central operating theatre keeps no records but the consumption pattern for six smaller theatres was established through requisition records kept in each. Following examples in the literature on allocating drugs expenditures across hospital cost centres [14], the unaccounted for receipts by the central operating theatre were assumed to be consumed directly there.

For the special clinic, some drugs were obtained from the main pharmacy and it was attempted to capture this consumption by the methods described above. In addition, the special clinic privately procures drugs and medical supplies. A computerised database includes drugs and medical supplies dispensed per patient, although it is not possible to classify patients by service unit (inpatient and outpatient) using this database. Instead, these costs were apportioned using inpatient equivalents calculated as patient days for the clinics involving the assumption that patient days are equivalent to attendances divided by the average length of stay (3.47 days in the special clinic).

#### Capital costs

Annual capital costs were collected from hospital level data and apportioned according to an inventory of furniture and movable equipment, valued at replacement cost. The estimated allocation to each service unit was annuitised using a discount rate of 5%. Rent values based on floor space were used to value buildings, many of which have no book value if a 30-year life is assumed.

Using the allocation of total cost to final cost centres which in turn had used a combination of step-down and bottom-up costing methods, unit costs were calculated on the basis of the activity statistics inpatient days, admissions and outpatient visits. Costs for intermediate cost centres' outputs: images, clinical tests, theatre cases, blood units, physiotherapy treatments and pathology tests were also calculated. The hospital's health information system database (computerised) was the main source of these data although in some cases supplementary data were collected from individual service units.

In the process of allocating costs to the special clinic, we encountered a number of difficulties. All the human resources costs borne by the clinic are accounted for in the clinic's books and paid directly to the MCH personnel as extra salary. Some special clinic costs like water and electricity bills are paid by the MCH and reported in the main hospital accounting books only. The clinic also purchases other specific goods and services (e.g. blood bags) from the hospital and these are paid directly to the hospital department/unit and reported in both the clinic and department books. Renting of MCH facilities and equipment and occasional drug supplies from the hospital to the clinic are not reported or accounted for.

Many MCH regular staff work extra hours and provide occasional services for the clinic. Although such costs are accounted for by the clinic, these are never discounted by the MCH side, whether they took place outside the hospital regular shift hours or not. Within the clinic very few workers are assigned exclusively to a specific department, and personnel costs had to be apportioned according to inpatient equivalents.

The effect of these limitations in our ability to track resource flows through the hospital is that costs of the special clinic measured exclude a number of allocations of shares of costs incurred by MCH. To the extent that these do not imply increased resource use in MCH, they imply a measure of cost close to average incremental cost. In other respects, where MCH costs are directly impacted by the special clinic, they understate the average incremental cost.

## Results

Tables 3 and 4 show selected unit costs by inpatient service unit for MCH and all unit costs by service unit for the special clinic respectively. The selected costs for MCH represent the range of costs and the different departments of the hospital.

**Table 3:** Selected unit costs by inpatient service unit, MCH ('000 meticais, FY2001)

Department Service Unit	Cost per admission	Cost per patient day
Department of Medicine		
General medicine ward 1	2,204	362
General medicine ward 3	1,348	240
Neurology	17,459	396

Department of Surgery		
General surgery	15,889	1,414
General abdominal	2,264	534
Urology	4,478	498

Department of paediatrics		
Short duration	275	413
Neonatal care	1,231	228
Malnutrition	2,414	205

Department of orthopaedics		
Male orthopaedics 1	6,603	280
Female orthopaedics	5,482	290
Paediatrics	3,017	316

Obstetrics and Gynaecology		
General obstetrics	915	681
Gynaecology	2,301	285

(M'000, FY2001)

**Table 4:** Special clinic: unit costs by inpatient service unit (M'000, cost excluding drugs and capital in parentheses)

Service Unit	Cost per admission	Cost per patient day
Clinic Sur	9,771 (9,318)	5,235 (4,992)

Clinic I	25,054 (23,650)	4,956 (4,678)

Clinic II	18,684 (17,450)	5,032 (4,705)

Clinic III	39,029 (35,593)	5,020 (4,707)

Currency: M'000, FY2001.

The differences between both types of unit costs in the two tables were marked and significant (p < 0.05 for admission; p < 0.001 for patient day). Such a crude comparison ignores case mix differences, which are very difficult to control for. Given the smaller number of service units, the special clinics service units catered for a heterogeneous mix of patients. Except in Clinic Sur (Emergency and Resuscitation) where patients tend to stay for a shorter period and are rapidly transferred, costs per admission were higher than even the most expensive specialist service units in MCH: neurology and general surgery. Even Clinic Sur costs per admission were higher than all but 3 specialist service units in MCH. (Costs per admission for Oncology in MCH, not included in Table 3, were 11,141,000 meticais.) Cost per patient day is everywhere in the special clinics at least four-fold the cost per patient day in any specialist unit of MCH.

A similar comparison is made between outpatient costs in Table 5.

**Table 5:** Cost per outpatient visit: MCH

Service unit	Cost per outpatient visit
Medical outpatients	48

Surgical outpatients	92

Paediatric outpatients	56

Orthopaedic outpatients	84

Obstetrics and gynaecology outpatients	95

Special clinic	1,302

Currency: M'000, FY2001.

The special clinic cost per outpatient visit was over 13-fold the cost for the highest cost service unit of MCH. That costs were significantly higher was expected given the expected difference in amenity levels between the two services. However, differences were greater, especially in the outpatient and patient day comparisons than easily explained by amenity alone, or by potential case mix differences.

There was also a marked difference in the share of capital and recurrent costs in the total between MCH and the special clinic. The total cost for the Special Clinic was 50bn meticais of which 98% was recurrent expenditure and 2% capital. In contrast, MCH had a more typical capital and recurrent cost balance. Of a total of M232bn, 85% was recurrent and 15% capital. The low level of capitalisation of the special clinics reflects its policy of outsourcing (largely from MCH) capital intensive services such as diagnostics and theatre, and is suggestive of the extent to which we have under-estimated the special clinic's use of these.

Table 6 compares the breakdown of recurrent costs between MCH and the special clinic.

**Table 6:** Recurrent costs by category, MCH and special clinic (million meticais)

Recurrent cost category	MCH total cost (% share)	Special clinic total cost (% share)
Personnel	65,130 (33)	35,953 (73)

Drugs	69,479 (35)	1,910 (4)

Medical supplies	23,756 (12)	2,260 (5)

Goods	29,808 (15)	4,984 (10)

Services	9,020 (5)	3,989 (8)

TOTAL	197,193 (100)	49,095 (100)

While a similar share of recurrent costs is accounted for by medical supplies, goods and services, the special clinic uses 73% of its recurrent resources for staff costs, and only 4% for drugs whereas MCH spends approximately equally on the two items. One explanation of this difference is the much higher expenditure on personnel in absolute terms in the special clinic. 61% of staff costs were physician costs. Physicians are mostly employed by the special clinic on a contract basis, and mostly paid on a per-visit or per-patient basis. Most of these physicians are full time employees of MCH but earn much more per hour on contract to the special clinic than through their MCH salaries. Despite the huge difference in the proportionate expenditure on drugs, special clinic patients still receive on average a higher value of drugs per inpatient day equivalent (M106,000) than MCH patients (M87,000). However these amounts are so small that it seems likely that special clinic patients' consumption has been under-estimated for reasons discussed above. Widespread unavailability and under provision of drugs in MCH is well known but is not a concern in the special clinic.

Table 7 shows costs of the extent of use of MCH services that we were able to trace. The total is small and amounts to approximately 2% of total special clinic expenditure. The special clinic makes a direct contribution to the income of MCH which amounts to M3.6bn, or 7% of total special clinic expenditure. In addition to this, certain ex-gratia payments are made to heads of sections and heads of nursing services, although these amount only to about M750 m (about 1.5% of total special clinic expenditure).

**Table 7:** Special clinic non-salary recurrent costs borne by MCH

Recurrent cost category	Cost (M'000)
Water and electricity	93,314

Maintenance of infrastructure	47,554

Imaging	164,600

Physiotherapy	16,514

Morgue	1,270

Theatre	418.233

Laboratory	98.027

Pathology	39,036

Blood	54,084

TOTAL	932,633

Currency: M'000, FY2001.

At the time of the study the special clinic was charging 520,000 MT (25USD) for an outpatient visit and 4,000,000 MT (200USD) for day spent in hospital, while the main hospital was either free for patients referred from other institutions or charging a "congestion fee" of 50,000 MT (2.3USD) per patient. The implication of this was that the clinic's marginal revenues were set below the average incremental costs shown above, which are themselves likely underestimates.

## Discussion

Despite the care that was taken to measure costs throughout the hospital and the special clinic, it seems that hospital records do not allow for the full identification of the use of MCH resources by the special clinic. A comparison of average incremental cost with marginal revenue indicates that the special clinic is cross-subsidised by MCH.

Furthermore, the estimates provided are almost certainly underestimates of the average incremental cost. A 2% rate of capitalisation is not viable for the well-functioning service that the special clinic appears to be. Although out-sourcing is a potential explanation and means of translating capital into recurrent cost, tracing costs through the major supplier of out-sourced services MCH, should translate those back to capital costs if the use of MCH made by the special clinic had been fully captured. Case-mix and focus on human resources-intensive activities like outpatient visits could help explain the relatively small share of the clinic's capital costs.

One review of hospital costing studies in developing countries suggests capitalisation levels (capital cost as a share of total cost) ranging from 17.8 to 43.8% where capital costs were thought to have been fully accounted for [^15^]. A study of 6 hospitals in Malawi measured capitalisation levels between 47 and 57% though used a different methodology from that used here, and may have overestimated the rate [16]. A study by one of the present authors in Zimbabwe, using an identical methodology to that used here and found capitalisation rates between 36 and 59% across 6 hospitals [17]. The capitalisation rate can vary widely across studies according to method of cost accounting and in cases of severe under-investment over a long period can drop well below the rates cited above. For example, a study of Niamey national hospital in Niger found a rate as low as 5% in a context where '*relatively little capital equipment...is operational' *[18]. Nevertheless, this is quite unlike the situation of the special clinic and we consider the 18% figure, the lowest of those compared across a range of settings where the methodology was judged sufficiently sound, the lower bound of a realistic capitalisation rate here. On that basis, there is approximately a shortfall of at least M8bn in the capital resource use of the special clinic that we have been able to account for. This significantly exceeds the full extent of the transfer payment from the special clinic to MCH. That payment would allow for a capitalisation rate of only 10.5%.

We also suspect that the special clinic's use of MCH's pharmaceutical stocks has been underestimated. The costing studies cited above can also be used to compare the expenditure on drugs as a share of recurrent costs, none reaching anything like as small a share as the 4.1% measured for the special clinic. The range for medical supplies as a whole across developing country settings was 10.3 to 32% [13], and for the six Malawian hospitals, 24.3 to 37.4% [14]. However, there are other possibilities than that any deficit in our drug use estimates have been at the expense of the MCH pharmacy. It is possible that we have failed to trace the special clinic's own expenditures on drugs, or that special clinic patients commonly take prescriptions to private pharmacies rather than receive and be billed for drugs in the clinic itself.

We face considerable difficulty in deciding how to treat the large expenditure on physician salaries. The above analysis treats these as cost. In principle, we seek to measure the opportunity cost of the resources used. Where these resources are traded in a competitive market, it is reasonable to treat price as equivalent to opportunity cost. However, the market for physician labour is distorted. Since most doctors work for the Ministry of Health, the Ministry is close to being a monopsonist purchaser of physician labour and exerts undue influence on the going salary rate. This produces a downward pressure on salaries and implies that MCH salaries understate the opportunity cost of physician labour. In the special clinic, effective salary rates are much higher, whereas economic theory would predict that a slightly higher salary than the public sector rate would attract ample supply of labour. There are at least three possible explanations of this:

1. While doctors have no alternative employment opportunities to public sector jobs, agency problems in the health sector imply that their commitment to those jobs and effort levels may be low without loss of salary. In order to purchase commitment and effort that may be required by higher monitoring capacity in the special clinic, higher levels of salary may be required. This implies that the salary levels observed indeed reflect opportunity cost and suggests no adjustments to our estimates are necessary.

2. Doctors in MCH may be the effective principals of the special clinic. Hospitals may be viewed as 'physician's co-operatives' [19] or more generally doctors may be their dominant decision makers [20]. This suggests that the special clinic may be allowed to function only by permission of doctors, and may buy this consent through the payment of high salary levels there. In this case, the salary levels observed are not opportunity costs but economic rents, or transfer payments, which should not be considered part of the costs of operation. This perspective has significant implications for our estimates as 45% of total special clinic costs are accounted for by payments to physicians. However, even if we assume that the real opportunity cost of physicians is zero, this downward adjustment is insufficient to bring special clinics' average incremental cost as low as marginal revenues for outpatient visits. In other words, the price charged for outpatient visits does not cover the non-physician staff time and other inputs. Inpatient charges would cover non-physician costs and approximately 55% of physician costs which is more than the public sector salary (which we assume below opportunity cost) and less than the special clinic salary which may contain an element of economic rent.

3. Current salary levels in MCH may be below the reservation wage, or the minimum required to secure the services of doctors in the labour market. By subsidising those wages through payments for work in the special clinic, doctors work for MCH as a whole may be secured. On this argument, the amounts paid to doctors might be considered a subsidy to MCH, which could be added to the transfer payments already identified. We cannot be more precise about the extent of this potential subsidy without further knowledge of the reservation wage.

Similar arguments can be made in relation to the transfer payments paid to MCH including the ex-gratia payments made to certain heads of MCH departments. These can be considered payment to cover the special clinic's use of MCH resources including administration by these individuals, or they may be considered rents that do not reflect resource flows from MCH to the special clinic. In principle, they should be included in or excluded from special clinic cost accordingly.

## Conclusion

Cost differences between MCH and the special clinic are large, particularly when outpatient and costs per patient day are compared. The differences are of an order of magnitude that cannot be explained credibly by case mix difference. By far the greatest contribution to the cost difference is the physician salary component, if this is indeed an opportunity cost and reflects the cost of achieving commitment and effort from doctors. This reflects both higher application of physician hours and a higher rate of pay per hour. This in itself suggests that cost differences do not reflect differences in amenity levels alone.

The price charged for special clinic services does not cover the cost of those services if the full expenditure on physician salaries is considered part of that cost. For outpatients, the price charged is insufficient to cover even non-physician time and other inputs used. This implies that MCH subsidises the special clinic, and that the government subsidy provided to MCH leaks to the support of the special clinic. If the physician salaries are instead interpreted as economic rents, the special clinic arrangement enables physicians to capture a share of the subsidy provided by the government to the MCH, presumably at the expense of patients there. Either way, it would appear that the special clinic imposes costs on users of MCH.

We were unable to capture fully the extent of special clinic use of resources in MCH. Those captured totalled approximately M1bn, only 2% of total special clinic resource use. The extent to which this represents an under-measurement of resource use is indicated by the low resulting measured level of capitalisation of the special clinic, also amounting to approximately 2%. A more likely minimum level of capitalisation, equal to that of MCH itself of 15% suggests a minimum resource flow from MCH to the special clinic of M6.5bn (assuming this resource flow was 100% capital) or M13bn (assuming a 50-50 split). It is likely that we also under-estimated special clinic patients' use of the MCH pharmacy.

Despite the attempts to separate sites and management, there is still considerable difficulty in accounting for the flow of resources between the main hospital and the clinic, especially of human resources and capital goods. There is a need to develop accounting systems that ensure greater transparency in order to establish that arrangements in the special clinic make the net contribution to public sector services for the majority they often claim to make. In the absence of such a transparent system demonstrating an equity promoting cross-subsidy, it is unsurprising that popular disapproval threatens the continuing operation of these arrangements.

## Competing interests

The authors declare that they have no competing interests.

## Authors' contributions

BMP participated in the design of the study and wrote the manuscript. CH participated in the design of the study, carried out data collection and analysis. GR participated in the design of the study, participated in data collection and edited the manuscript. All authors read and approved the final manuscript.

## Pre-publication history

The pre-publication history for this paper can be accessed here:

http://www.biomedcentral.com/1472-6963/11/143/prepub

## References

[B1] Shirom A Private medical services in acute-care hospitals in Israel International Jounal of Health Planning & Management 2001 16 325 45 10.1002/hpm.642 11771151

[B2] McPake B Hanson K Adam C Two-tier charging in public hospitals: implications for intra-hospital resource allocation and equity of access to hospital services Journal of Health Economics 2006 26 3 447 462 17147962 10.1016/j.jhealeco.2006.10.011

[B3] Wadee H Gilson L Blaauw D Erasmus E Mills A Public-Private Interactions in the South African Health Sector: Experience and Perspectives from National, Provincial and Local Levels 2001 Wits University, Johannesburg Report for the Local Government and Health Consortium

[B4] Nakamba PM Hongoro C McPake B Hanson K Eriksson P, Diwan V, Karlberg I Country case study: Zambia Health Sector Reforms: What about hospitals? 2002 Nordic School of Public Health 2002: Gotenborg, Sweden

[B5] Ferrinho P Lerberghe W Fronteira I Hipólito F Biscaia A Dual practice in the health sector: review of the evidence Human Resources for Health 2004 2 14 10.1186/1478-4491-2-14 15509305 PMC529467

[B6] GoM Law no. 26/91 of August 16 1991 Boletim da Republica

[B7] MoH Ministerial Decree No. 09/92 of May 26 1992 Gabinete do Ministro da Saúde Maputo

[B8] Muchanga S Arao E Pedro PF Povo aplaude decisão de Garrido Savana 2007 access online on 16.03.2007

[B9] Jornal Notícias A partir deste ano: Clínicas especiais vão sair dos hospitais públicos 2007 Portal do Governo de Moçambique http://www.portaldogoverno.gov.mz/noticias/news_folder_sociedad_cultu/fevereiro2007/nots_sc_116_fev_07/

[B10] Vinyals L Estudo dos custos da cirugia no Hospital Central de Maputo Livro branco da cirujia 2000 Consejo Espanol de Cooperacción, Maputo

[B11] Russo G Polonia E Munguambe E Estudo sobre custos da prestaçãoo de serviços de saúde de atenção primaria em Moçambique 2000 MISAU and Austral, Lda, Maputo

[B12] Conteh L Walker D Cost and unit cost calculations using step-down accounting Health Policy Plann 2004 19 127 135 10.1093/heapol/czh015 14982891

[B13] Oostenbrink JB Koopmanschap MA Rutten FFH Standardisation of Costs: The Dutch Manual for Costing in Economic Evaluations Pharmacoeconomics 2002 20 7 443 454 10.2165/00019053-200220070-00002 12093300

[B14] Litvak J Hospital cost analysis: allocating pharmaceutical expenditures in Papua New Guinea Health Policy Plann 1991 6 1 71 77 10.1093/heapol/6.1.71

[B15] Mills A The economics of hospitals in developing countries--Part 2: Costs and sources of income Health Policy and Planning 1990 5 3 203 18 g 10.1093/heapol/5.3.203

[B16] Mills AJ Kapalamula J Chisimbi S The cost of the district hospital: a case study in Malawi Bulletin of the World Health Organisation 1993 71 3-4 329 39 PMC2393502 8324852

[B17] Hongoro C Costs and quality of services in public hospitals in Zimbabwe: implications for hospital reform 2001 Thesis (PhD) -- University of London

[B18] Weaver M Wong H Sako AS Simon R Lee F Prospects for reform of hospital fees in sub-Saharan Africa: A case study of Niamey National Hospital in Niger Social Science & Medicine 1994 38 4 565 574 10.1016/0277-9536(94)90253-4 8184319

[B19] Pauly M Reddisch M "The Not-For-Profit Hospital as a Physician's Cooperative." The American Economic Review 1973 63 1 87 99

[B20] Strong P Robinson J New model management: Griffiths and the NHS 1988 University of Warwick Nursing Policy Studies Centre; 3

